# Comparison of Hard Tick (Acari: Ixodidae) Fauna in Natural and Anthropogenic Habitats in Croatia

**DOI:** 10.3390/insects16101027

**Published:** 2025-10-05

**Authors:** Stjepan Krčmar, Marko Vucelja, Marco Pezzi, Marko Boljfetić, Josip Margaletić, Linda Bjedov

**Affiliations:** 1 Department of Biology, Josip Juraj Strossmayer University of Osijek, Cara Hadrijana 8/A, 31000 Osijek, Croatia; stjepan@biologija.unios.hr; 2 Faculty of Forestry and Wood Technology, University of Zagreb, Svetošimunska cesta 23, 10000 Zagreb, Croatia; 3 Department of Chemical, Pharmaceutical and Agricultural Sciences, University of Ferrara, Via Luigi Borsari 46, 44121 Ferrara, Italy; marco.pezzi@unife.it

**Keywords:** parasitiformes, Ixodida, diversity, Bansko Hill, Medvednica Mountain, Croatia, Europe

## Abstract

**Simple Summary:**

In response to increasing global concern regarding the occurrence of hard ticks (Acari: Ixodidae) and tick-borne diseases, we investigated the diversity, structure and seasonal dynamics of hard tick fauna in natural habitats (NHs) (i.e., pedunculate oak, common beech and silver fir forest communities, 200–1000 m a.s.l.) and anthropogenically conditioned habitats (AHs) (i.e., orchards, grasslands, degraded forests, 150–250 m a.s.l.) in Continental Croatia (2019–2021, 2023–2024 yr.). Flag-dragging method was used to sample host-seeking ticks, resulting in the identification of 2726 specimens from AHs and 1543 hard ticks sampled in NHs. The AHs showed a greater species diversity, with eight tick species identified (*Ixodes ricinus*, *I. frontalis*, *I. hexagonus*, *I. kaiseri*, *Haemaphysalis inermis*, *H. concinna*, *Dermacentor marginatus*, *D. reticulatus*) compared to only three (*I. ricinus*, *I. frontalis*, *D. reticulatus*) in the NHs. The most abundant species in both environments was *Ixodes ricinus*, one of the major tick-disease vectors, which exhibited a bimodal seasonal activity in AHs and a unimodal pattern in NHs. Abundance of some species, such as *Haemaphysalis inermis* and *Dermacentor reticulatus*, showed a significant negative correlation with temperature and a positive correlation with humidity in AHs. Conversely, *I. ricinus* abundance in NHs was positively associated with temperature and negatively with humidity. The study also documented new distributional records, including *I. frontalis* in eastern parts of the country and *D. reticulatus* at a new elevation of 1000 m above sea level in Central Croatia. These findings contribute new data on the distribution and seasonality of medically important tick species, aiding in the identification of tick-risk foci and high-risk periods.

**Abstract:**

Due to the evident increase in tick-borne diseases worldwide, it is necessary to constantly update information on the distribution and zoonotic potential of hard ticks. We studied diversity, population structure, and seasonal dynamics of hard tick fauna, faunal similarity and the climate impact on tick occurrence in natural habitats (NHs) (forest communities) and anthropogenic habitats (AHs) (orchards, grasslands, degraded forests) in eastern and central parts of Continental Croatia. Host-seeking hard ticks were sampled by the flag-dragging method in lowland AHs (Bansko Hill (BH); 2023–2024 yr.) and in mountainous NHs (Medvednica Mountain (MM); 2019–2021, 2024 yr.). Overall, 2726 specimens belonging to eight hard tick species (*Ixodes ricinus*, *I. frontalis*, *I. hexagonus*, *I. kaiseri*, *Haemaphysalis inermis*, *H. concinna*, *Dermacentor marginatus*, *D. reticulatus*) were identified in AHs, while in NHs 1543 hard ticks, belonging to three species (*I. ricinus*, *I. frontalis*, *D. reticulatus*), were collected. The most abundant species in both habitat types (47.83% in AHs, 99.80% in NHs) was *I. ricinus*, showing unimodal seasonal activity within studied NHs and bimodal activity at AHs. Comparison of hard tick fauna in different habitats using the Sørenson index on BH and MM showed a high percentage of similarity (50.0–88.8). At AHs, a significant (*p* < 0.05) negative correlation was determined between the abundance (*N*) and the mean monthly air temperatures (°C) for *H. inermis* (*r* = −0.5931; *p* = 0.0421) and *D. reticulatus* (*r* = −0.6289; *p* = 0.0285), while their numbers positively correlated (*r* = 0.5551; *p* = −0.2667; *r* = 0.4430; *p* = 0.1492) with air humidity (%). In contrast, the number of sampled host-seeking *I. ricinus* ticks at natural forest habitats on MM was positively associated with air temperature and negatively with air humidity at elevations from 200 to 1000 m a.s.l. (*r* = −0.7684; *p* = 0.0259; at 200 m a.s.l.). Collected specimens of *I. frontalis* mark the first record for Osijek–Baranja County, while the sampled *D. reticulatus* on MM represents the first catch at 1000 m a.s.l. in Croatia. This new data on the distribution and seasonality of medically important hard tick species in Continental Croatia contributes to identifying tick-risk foci and high-risk periods.

## 1. Introduction

Globally, hard ticks are the subject of extensive research in medical and veterinary parasitology, as they serve as vectors for various pathogenic microorganisms, including bacteria, viruses, protozoa, and helminths [1]. Even though only approximately 10% of the currently known tick species act as vectors of pathogens [2], ticks and tick-borne diseases are an increasing concern worldwide, with the (re)emergence of diseases affecting human and animal health [3,4]. However, the factors driving the increase in disease in certain areas, along with the emergence of new outbreaks where the pathogen was previously absent, are quite varied and often local [3]. Changes in vertical and horizontal range of tick species and the diseases they carry have been reported locally and worldwide [3,4,5]. The main factors for the emergence and spread of ticks and their pathogens—in both animals and humans—are connected mainly by progressive climate changes [6] and the changes in the landscape derived from a series of human activities such as globalization of the economy, urbanization, deforestation, habitat changes, changes in agricultural land use, as well as increased human and animal migration [7,8,9]. As tick-borne diseases are becoming more prevalent worldwide, there is a clear need for continuous monitoring of tick fauna to track their presence, distribution, and the prevalence of associated pathogens [3,10]. Europe, in particular, has seen a notable expansion of tick-borne zoonoses, with tick-borne encephalitis (TBE) and Lyme disease showing a marked increase [11,12,13]. Furthermore, some important ecological issues regarding “habitat–tick co-existence” still need to be answered. For instance, while the connection between the biodiversity of the habitat (or hosts) on one hand, and ticks and tick-borne pathogen abundance on the other, have been discussed earlier [14,15], there are still doubts whether that relationship is always inverse, without exception. In Croatia, research on tick diversity and vector potential has previously predominantly focused on the Mediterranean and northwestern regions [16,17], with more recent studies extending to Eastern and Central Croatia [18,19,20]. Despite that, many areas in Croatia have not yet been sufficiently studied, especially anthropogenic habitats. Bansko Hill (BH) in the Croatian part of Baranja represents such a habitat type. The northeastern side of BH is covered with secondary forest vegetation, and serves as a refuge for large game, while the southwestern side is characterized by various agro-ecosystems (orchards, vineyards, wheat fields), degraded semi-natural grasslands and small remnants of degraded forests. Both sides are subjected to significant anthropogenic influences, including hunting tourism, recreational activities such as the Baranja Night Trail, and agricultural practices. During these activities, humans and their pets are often exposed to hard tick bites. Another notable location, drawing approximately one million visitors each year [21], is the Medvednica mountain (MM) massif, situated just north of Croatia’s capital, Zagreb. It is visited for its floral and faunal biodiversity [22], but is also identified as a Lyme disease and tick-borne encephalitis focus [23]. This research primarily aimed to investigate the diversity of hard ticks on different anthropogenic habitats (AHs) at BH and compare it with those found in the natural habitats (NHs) of MM. The further goals of this research included evaluation of the potential association between certain tick species and specific habitat types, analysis of the abundance and seasonal dynamics, faunal similarity and climatic conditions assessment.

## 2. Materials and Methods

### 2.1. Study Area

**Figure 1:**
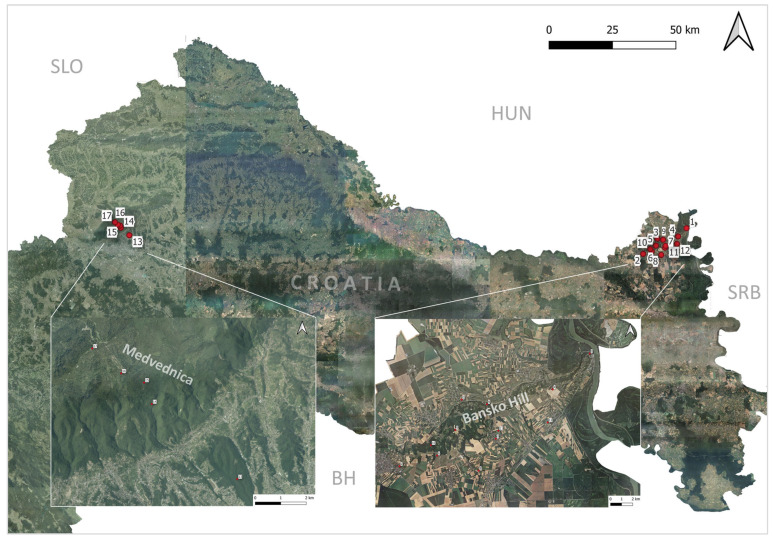
Hard-tick sampling sites at Bansko Hill and Medvednica Mountain within the Continental biogeographic region in Croatia. 1—Batina; 2—Beli Manastir; 3—Branjina; 4—Draž (Vidikovac-Trojnaš); 5—Kamenac (Odašiljač Belje); 6—Karanac (Vidikovac Belje); 7—Kotlina; 8—Kneževi Vinogradi; 9—Podolje; 10—Popovac (Rudnik); 11—Suza; 12—Zmajevac; 13—Medvednica Mountain 200 m a.s.l.; 14—MM 400 m a.s.l.; 15—MM 600 m a.s.l.; 16—MM 800 m a.s.l.; 17—MM 1000 m a.s.l.

Bansko Hill (BH) (Figure 1) is a loess elevation extending in a northeast-southwest direction for approximately 21 km long, with a significantly smaller width of about 3 to 4 km, and its highest peak reaching 243 m above sea level [24]. This hill represents the highest relief feature in the Croatian part of Baranja. On the southeastern side, erosional processes caused by the Danube River have formed loess cliffs ranging from 25 to 58 m in height [25]. Erosion on the plateau of BH has also created ravines and loess valleys, which predominantly run in a northwest-southeast direction [24,25]. The core of BH consists of Miocene marl, limestone, sandstone, conglomerates and clay, with occasional intrusions of basalt-andesite [26]. These rocks are generally covered by sandy loess deposits [26]. The northeastern side of BH is covered with secondary forest vegetation dominated by black locust (*Robinia pseudacacia* L.), walnut (*Juglans regia* L.), linden (*Tilia* sp.), tree of heaven (*Alianthus altissima* Mill. Swingle) and hornbeam (*Carpinus betulus* L.), while the shrub layer primarily features elderberry *(Sambucus nigra* L.), hawthorn (*Crataegus monogyna* Jacq.) and dogwood (*Cornus mas* L.). These are lighter forest communities characterized by substantial sunlight penetration, allowing the growth of dense understory vegetation, including common nettle (*Urtica dioica* L.), false indigo (*Baptisia* sp.) and greater celandine (*Chelidonium majus* L.), which entirely cover the forest floor. On the southwestern side, the landscape is predominantly composed of various agro-ecosystems, degraded semi-natural steppe grasslands, and small remnants of xerothermic forests with sporadic occurrences of downy oak *Quercus pubescens* Willd. [25]. The climate of BH is moderately continental, belongs to Cfb temperate climate type according to Köppen, with an average annual temperature between 10 and 11 °C and average annual precipitation ranging from 600 to 700 mm [24]. There are two precipitation maxima: one at the end of spring and the beginning of summer, and another in autumn, giving the area a sub-Mediterranean climate characteristic [24,25].

Located at the rim of the Pannonian Plain, Medvednica (Figure 1) is a separate mountain (Medvednica Mountain = MM) massif that forms a prominent relief within a wider range of the City of Zagreb [27]. It stretches (42 km in length) in a northeast–southwest direction, with the highest peak Sljeme (1035 m above sea level). With a protected area of 228 km^2^, MM was declared a Nature Park in 1981. The steep and inaccessible slopes, covered with dense and homogeneous forests, dominate the landscape. Meadows and grasslands cover a very small proportion of the peak zone (including ski resort) and eastern parts of the Park, while agricultural areas can be found on the northern side [28]. In surrounding landscape urban, suburban and rural settlements intertwine with agricultural land (orchards, vineyards, arable land) and isolated groves. Medvednica Mountain is primarily (63%) covered in natural forests, ranging from sessile oak (*Quercus petrea* L.) in the lower parts, through beech forests (*Fagus sylvatica* L.) to beech and fir (*Abies alba* Mill.) forests in the highest areas [29]. Rich diversity of forest communities is a result of climatic features, indented relief, various geomorphological characteristics, geological substrates and soil types. As forests occupy the largest area size on MM, five target habitat types include (a) acidophilic beech forests (*Luzulo-Fagetum*); (b) mixed forests (*Tilio-Acerion*) of maple (*Acer pseudoplatanus* L.), ash (*Fraxinus* sp.), elm (*Ulmus* sp.) and linden (*Tilia platyphyllos* Scop., *T. cordata* Mill.) [30]; (c) sweet chestnut (*Castanea sativa* Mill.) forests within a mixed forest community of sessile oak (*Quercus petrea* L.) and sweet chestnut; (d) Illyrian *Fagus sylvatica* L. forests [22] (with Blagay daphne (*Daphne blagayana* Freyer), the laurel daphne (*Daphne laureola* L.), the holly (*Ilex aquifolium* L.), the broad-leaved ruscus (*Ruscus hypoglossum* L.) and the white udder (*Platanthera bifolia* L.) present in the shrub and ground layer; and (e) Illyrian oak-hornbeam forests (*Erythronio-carpinion*), [31]. The pedosphere of this area is closely related to the lithological base [32], and the most common are acid brown soils (Dystric Cambisol). More than 2000 taxa (species and subspecies) have been recorded in the entire study area, where invertebrates and plants (around 1400 sp.) and birds (around 96 sp.) are the most numerous, while 249 species have a certain protection or the endangered species status [22,33]. Medvednica Mountain belongs to Cfb—a temperate climate type according to Köppen, without a dry season and with a warm summer [34]. The coldest month of the year (January) temperature is above −3 °C, while the summers are fresh, with the average monthly temperature of the hottest month being below 22 °C [35].

### 2.2. Field Work and Identification of Sampled Ticks

Tick sampling on Bansko Hill (BH) was conducted from September 2023 to December 2024. During the autumn, winter, and spring sampling occurred five times per month, while in summer it was conducted twice a month. Samplings were carried out at 12 localities, four on the northeastern and eight on the southwestern side (Figure 1, Table 1). All 12 sampling sites at BH were located outside the human settlements, some up to five kilometers away. Localities near the settlements of Zmajevac, Suza and Kotlina are small mixed degraded forests (shrubland), overgrown with black locust, tree of heaven, walnut, linden and downy oak. Sampling sites in the area of the settlements of Batina, Kneževi Vinogradi, Draž (Vidikovac Trojnaš) and Podolje were in untended orchards. Localities in the area of Kamenac (Odašiljač Belje), Karanac (Vidikovac Belje), Popovac (Rudnik), and Beli Manastir are covered mainly with black locust trees. Only the locality in Branjina was overgrown with semi-natural grassland vegetation with forest vegetation of black locust and walnut on the edges.

From 2019 to 2021 sampling of hard ticks on MM was carried out twice a year (spring and autumn), while during 2024 it was performed once a month, from spring till autumn. Tick sampling included five localities on different altitudes (200, 400, 600, 800, 1000 m a.s.l.) with three different forest community types (pedunculate oak forest, European beech and Pannonian beech–fir forest) (Table 1).

Data on the forest communities was obtained by “Hrvatske šume” (Croatian Forests), a state-owned enterprise responsible for woodland management in the Republic of Croatia. The nomenclature of forest communities corresponds to “Forest phytocenology and forest communities in Croatia” [29] and “Croatian forest vegetation” [36]. The list of sampling sites, their GPS positions, altitudes and habitat types (e.g., forest communities, orchards …) are shown in Table 1. Ticks were collected using the flag-dragging method [37,38], with white flannel cloth (1 m × 1 m) that was pulled over the ground surface and the vegetation and inspected every 5–10 m, depending on the catch. At BH and MM, each sampling session lasted approximately 30 min per locality. Ticks were collected from the flag with tweezers, stored in plastic vials (tubes) (Eppendorf, 1.5 mL) with a safety cap and preserved in a 96% ethanol solution or in a freezer (−80 °C). The identification of the collected ticks was carried out according to keys [39,40,41], and illustrations for species identification [42]. For the determination of ticks, a Leica Wild Stereo Microscope MZ8 (Leica Microsystems, Mannheim, Germany) light microscope was used (magnification 50×) equipped with an object micrometer together with the Quick Photo software package, ModellCamera 2 and a Dino-Lite digital microscope (magnification 20×–220×; 500×) (AnMo Electronics Corporation, Taiwan, China) with DinoCapture 2.0 software, version 1.5.17. The fieldwork was carried out under permits issued by the Ministry of Economy and Sustainable Development of the Republic of Croatia (UP/I-612-07/19-48/154, 517-05-1-1-19-3, 26.06.2019). All ticks collected on Medvednica Mountain (MM) are deposited in the tick collections of the Faculty of forestry and wood technology, University of Zagreb, while 1746 ticks collected on Bansko Hill (BH) were sent to the Croatian Veterinary Institute in Zagreb for pathogen analysis.

### 2.3. Faunal Similarity and Climatic Conditions Assessment

Analysis of tick fauna diversity among different vegetation types was conducted using the Sørensen faunal similarity index [43]. Observed differences in the number of ticks collected in different developmental stages were analyzed with the Chi-square test; *p* values < 0.05 were considered statistically significant. Climate assessment included data analyses of mean monthly air temperatures (°C), air humidity (%) and total precipitation (overall rainfall in mm) for meteorological stations closest to study sites (meteorological station Puntijarka for site MM; meteorological station Osijek for BH). Meteorological data were kindly provided by the Croatian Meteorological and Hydrological Service [44]. Climatic conditions on monthly/seasonal/annual scale are assessed by mean daily air temperature (°C) and precipitation (%) anomalies with respect to the corresponding 30-year reference climate period, and according to the associated percentile values [45]. The classification scale is used with limits determined according to the following percentile values: 2nd, 9th, 25th, 75th, 91st and 98th. The 1991–2020 normal has been in use since January 2023 (1981–2010 normal was used until then). The correlation between the abundance (N) of collected ticks and the average monthly weather conditions was tested with the Pearson’s correlation coefficient (r, *p* < 0.05) using the Statistica Version 14.1.0.8 TIBCO Software Inc. (Cloud Software Group, Inc. (2023), Fort Lauderdale, FL, USA. Data Science Workbench, version 14. http://tibco.com, San Ramon, CA, USA) [46]. Classifications of magnitude were interpreted as follows: 0.00 < 0.20 = very weak correlations; 0.20–0.39 = weak correlations; 0.40–0.59 = moderate correlations; 0.60–0.79 = strong correlations; correlations > 0.80 as very strong [47].

## 3. Results

### 3.1. Identified Tick Species

During multiple years of hard-tick sampling (2019, 2020, 2021, 2023, 2024) at 17 localities within the Continental biogeographic region (Figure 1, Table 1) in Croatia, overall, 4269 individuals belonging to eight species from three genera (*Ixodes*, *Haemaphysalis* and *Dermacentor*) were collected (Table 2). A total of 2726 (63.9%) hard ticks belonging to eight species were collected during 2023 and 2024 at 12 localities in the area of Bansko Hill (BH), where *Ixodes ricinus* was the most abundant with 47.83% of the collected ticks, followed by *Haemaphysalis inermis* with 42.07%, *Haemaphysalis concinna* with 8.5%, *Dermacentor marginatus* with 1.1%, *Dermacentor reticulatus* with 0.36% and three species, *Ixodes frontalis*, *Ixodes hexagonus* and *Ixodes kaiseri,* representing 0.10% of the collected specimens (Table 2). Between the localities at BH the total number of collected tick specimens varied from *N* = 33 (Batina) to *N* = 556 (Popovac) (Table 2). At five localities on Medvednica Mountain (MM) overall 1543 (36.1%) hard ticks, belonging to three species, were collected during the four-year sampling (2019, 2020, 2021, 2024), and the most abundant species was *I. ricinus* with *N* = 1540 (99.80%) specimens, followed by *D. reticulatus* with two individuals (0.13%) and *I. frontalis*, represented by a single catch (0.06%) (Table 2). The highest number of species (*N* = 6) was sampled at the Zmajevac locality, while at localities Beli Manastir and Popovac five species of ticks were identified (Table 2). At other localities, the number of collected species varied between two and four.

On BH, only two species, *I. ricinus* and *H. inermis* were collected in all localities, followed by *H. concinna* in nine, while other species were recorded in a fewer number of localities (Table 2). *Ixodes ricinus* was the most common species at the seven localities, while *H. inermis* was the most common at five (Table 2). Three species, *I. ricinus*, *H. inermis* and *H. concinna,* were collected in all vegetation communities in the study area at BH (Table 2). *D. marginatus* was absent in untended orchards, while *D. reticulatus* in semi-natural grasslands. Other three species, *I. frontalis*, *I. hexagonus* and *I. kaiseri,* were collected in habitats with one vegetation type (Table 2).

Within the research area on MM, *I. ricinus* was also recorded in all localities (Table 2), inhabiting the studied forest communities of pedunculate oak, European beech and panonian beech–fir forests at altitudes from 200 to 1000 m a.s.l. (Table 2)*. Ixodes frontalis* nymph was sampled once within Pedunculate oak forest with European hornbeam (*Carpino betuli—Quercetum roboris typicum* Rauš 1969).

**Figure 2:**
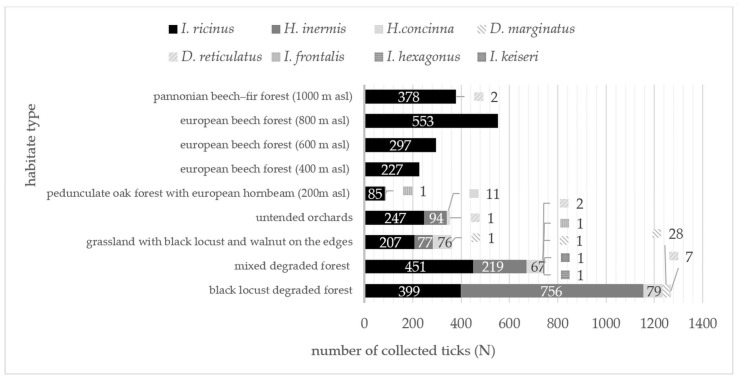
Total number of collected ticks, depending on the tick species and habitat type, sampled in NHs on Medvednica Mountain (2019–2021, 2024) and AHs on Bansko Hill (2024).

New locality records are provided for tick species collected at localities Batina, Beli Manastir, Kamenac, Karanac, Kotlina, Kneževi Vinogradi and Suza (Table 2), while the collected specimens of *I. frontalis* are among the first records in Eastern Croatia. Sampled male and female of *Dermacentor reticulatus*, collected from the Pannonian beech–fir forest (*Festuco drymeiae—Abietetum* Vukelić et Baričević 2007), represent the first record for MM at the altitude of 1000 m a.s.l. (Table 2). Overall number of collected ticks, depending on the tick species and habitat type, sampled at both study sites, are presented in Figure 2, showing the highest tick species variety at mixed degraded forest habitat on BH. The highest cumulative tick abundance recorded on BH was at the black locust degraded forest habitat and on MM within the European beech forest at 800 m a.s.l. altitude.

Out of all ticks collected at study site BH, the largest proportion (*N* = 1488; 54.58%) were in their adult stage, 1071 (39.29%) of the specimens were nymphs, while 167 (6.13%) sampled ticks were larvae (Table 3). *Ixodes ricinus* and *H. concinna* were the only two species collected at all developmental stages (Table 3). Five species (*D. marginatus*, *D. reticulatus*, *I. frontalis*, *I. hexagonus*, *I. kaiseri*) were collected only in the adult stage, while *H. inermis* was also represented in the nymphal stage (Table 3).

At study site MM, most of the collected ticks were nymphs, accounting for 962 individuals (62.35%), 324 (20.99%) were larvae and 257 (16.66%) were in the adult stage (Table 4). The only species collected at all developmental stages was *I. ricinus*, whereas both *D. reticulatus* specimens were sampled as adults (one female, one male) and *I. frontalis* as one nymph (Table 4).

**Figure 3:**
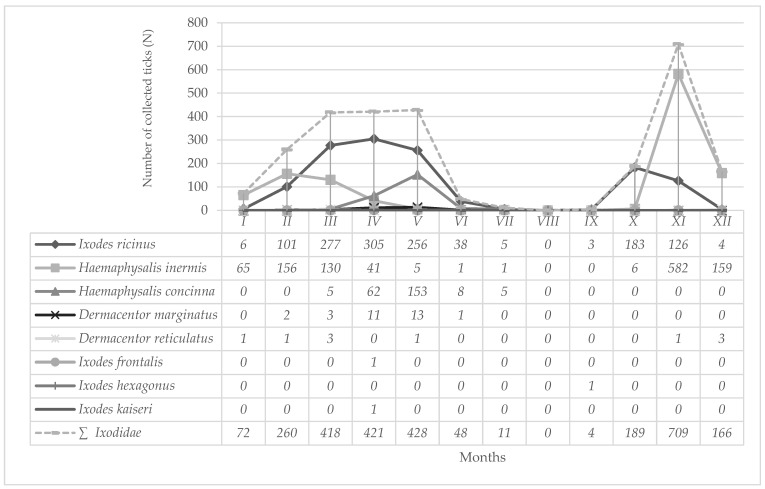
Monthly dynamics of hard tick species collected on Bansko Hill (2024).

Monthly dynamics of hard tick species collected at BH during year 2024 is presented in Figure 3. During spring months (March, April, May) on BH 46.47% of ticks were collected, while during autumn months (September, October, November) 33.08% of ticks were collected. Species *I. ricinus* showed bimodal seasonal activity with the first highest activity peak recorded in April and the second in October (Figure 3). Also, on BH bimodal activity patterns were recorded for *H. inermis*, with the first activity peaks in February and the second in November (Figure 3). For *H. concinna* and *D. marginatus* the highest number of individuals was recorded in May (Figure 3). Other tick species are not analyzed because of the small number of collected specimens.

**Figure 4:**
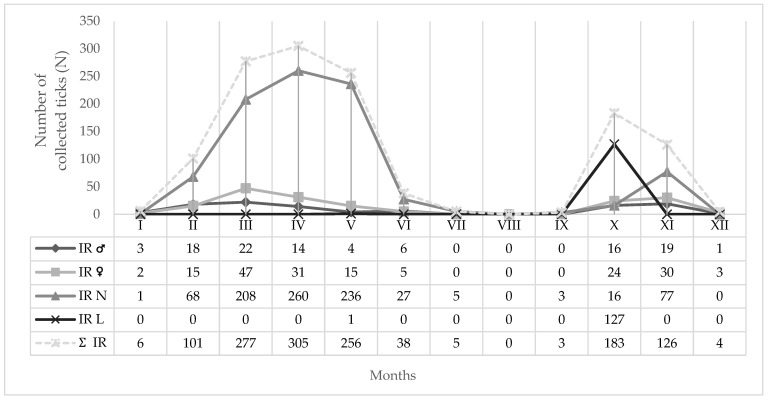
Monthly dynamics of IR = *I. ricinus* developmental stages (♂: male, ♀: female, N: nymph, L: larva) on Bansko Hill (2024).

Monthly dynamics of *I. ricinus* developmental stages (♂: male, ♀: female, N: nymph, L: larvae) recorded on BH are presented in Figure 4. *Ixodes ricinus* nymphs were the most abundant during the spring months (March, April and May) with 78.02% in the sample of collected nymphs (Figure 4). Larvae prevailed only in October with *N* = 127 specimens collected (Figure 4), while *H. concinna* was most abundant in the nymphal stage in April and May. The number of collected adults, nymphs and larvae of *I. ricinus* and *H. concinna* differed significantly at study site BH (χ^2^ = 775.42, *p* < 0.05; χ^2^ = 151.60, *p* < 0.05).

**Figure 5:**
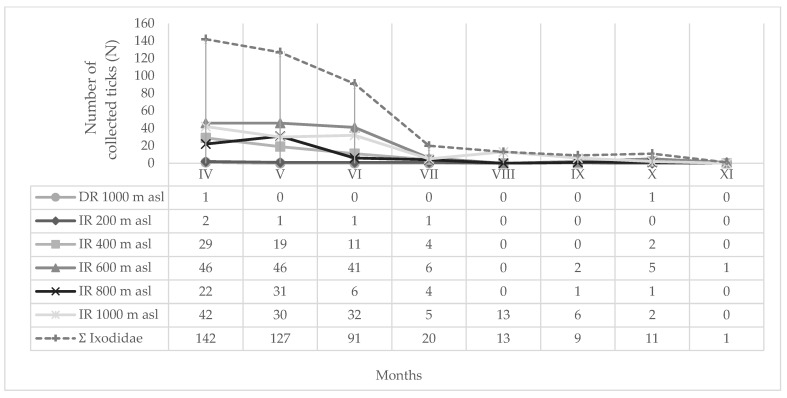
Monthly dynamics of hard ticks (IR = *I. ricinus*; DR: *D. reticulatus*) on Medvednica Mountain (2024) at different altitudes (200–1000 m a.s.l.).

**Figure 6:**
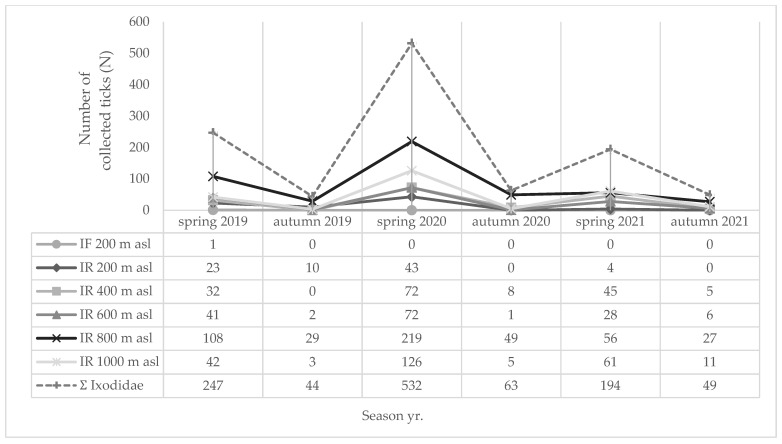
Seasonal dynamics of hard ticks (IR = *I. ricinus*; IF: *I. frontalis*) on Medvednica Mountain (2019–2021) at different altitudes (200–1000 m a.s.l.).

**Figure 7:**
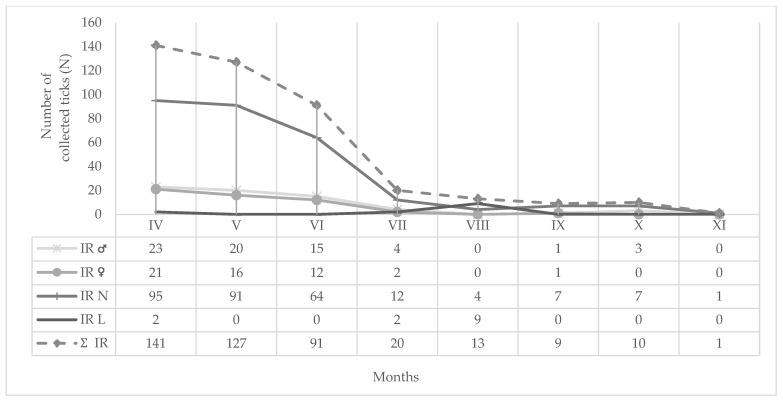
Monthly dynamics of IR = *I. ricinus* developmental stages (♂: male, ♀: female, N: nymph, L: larva) on Medvednica Mountain (2024).

Monthly (2024) and seasonal dynamics (2019–2021) of hard ticks (*I. ricinus*, *D. reticulatus*, *I. forntalis*) at different altitudes (200–1000 m a.s.l) on MM are shown in Figure 5, Figure 6 and Figure 7, respectively. During the spring months in 2024, 64.81% of hard ticks were collected (Figure 5), while 86.18% of hard ticks were sampled during the spring seasons in the period from 2019 to 2021 (Figure 6).

Observations on MM in 2024 revealed a unimodal peak in *I. ricinus* seasonal activity at 200 m a.s.l. and 1000 m a.s.l. A minor autumnal rise in collected tick numbers was also noted at altitudes of 400, 600 and 800 m a.s.l. (Figure 5).

#### 3.1.1. Faunal Similarity

The comparison of hard tick faunas recorded at BH in four vegetation types via the Sørenson index showed that fauna of black locust degraded forest and untended orchards as well as semi-natural grasslands were most similar to each other (88.88%) (Table 5). The lowest similarity was found between mixed forest communities and untended orchards and semi-natural grasslands (66.66%) (Table 5). In the same comparison on MM, the lowest similarity was observed between Pannonian beech–fir forests and Pedunculate oak forest with European hornbeam (Table 6). The similarity value between the other two forest communities was (66.66%) (Table 6).

#### 3.1.2. Correlation Analysis

The correlation between monthly weather conditions (mean air temperature (°C), relative humidity (%), and hard tick abundance (*N*) at study sites BH and MM, as determined by correlation analysis, is illustrated in Table 7 and Table 8.

## 4. Discussion

Being at the “crossroads” of Central and Southeast Europe, Croatia is a country well known for its biodiversity [48]. When discussing ticks and tick-borne diseases in Croatia, we need to take into account that woodland habitats are considered an ideal environment for hard tick fauna [49,50], and approximately half of Croatian land territory is covered with forests, making them the dominant land ecosystem [51]. Being previously aware that there are significant regional differences in host-seeking hard tick abundance and diversity in Continental, Alpine and Mediterranean biogeographic regions [20,52,53], we questioned the potential connection between certain hard tick species and specific habitat type by comparing the number of sampled specimens, seasonal dynamics and the weather conditions between anthropogenic habitats (AHs) (e.g., orchards, grasslands, degraded forests) with an uneven canopy cover and naturally preserved, homogeneous dense forest habitats (NHs) in Croatia inland.

Eight species from three genera of hard ticks (*Ixodes*, *Haemaphysalis* and *Dermacentor*) have been collected by the flag-dragging method in AHs at the study site Bansko Hill (BH) in Baranja, making this area one of the most diverse tick habitats in Croatia (Figure 1 and Figure 2; Table 2). These results, except for *I. canisuga*, almost match previous findings, where seven tick species were collected, also by flagging, from 2016 to 2018 at 48 localities in Eastern Croatia [53], indicating that prevailing ecological conditions and host availability within studied area support the recorded biodiversity and abundance of the present hard tick fauna. Earlier research conducted in different AHs and NHs in Baranja reports on five hard tick species, all matching our own results, but with the difference in three species from genus *Ixodes* (*I. frontalis*, *I. hexagonus*, *I. keiseri*) [54]. Another study, but from the western part of the country, that matches the number of hard tick species recorded at BH, was the one conducted by Cvek and collaborators [55], which included different types of AHs and NHs throughout Istria County, where seven hard tick species were collected, also using the flagging method. High biodiversity of hard tick fauna was also recorded at village Ponikve (Primorje–Gorski Kotar County, western Croatia), where four species of hard ticks from two genera (*Ixodes*, *Haemaphysalis*) were collected during a short period (March–June 2021) at AHs (cut grassland/mowed meadow) and NHs (mixed degraded forest of European hop-hornbeam (*Ostrya carpinifolia*), oriental hornbeam (*Carpinus orientalis*), downy (pubescent) oak (*Quercus pubescens*) and Montpellier maple (*Acer monspessulanum*) [56]. Of course, when considering results of different studies, the hard-tick sampling method’s importance should not be overlooked. For instance, the study from Cvek and collaborators [55] reported four additional species (12 hard tick species all together) when ticks—that were sampled from the animal hosts—are counted, while the study from Mičetić [56] counted five species in total, since one additional species from genus *Rhipicephalus* was sampled from domestic animal hosts.

The dominant hard tick species among collected specimens at AHs (47.8%) on BH and NHs on Medvednica Mountain (MM) (99.8%) was *I. ricinus* (castor bean tick) (Figure 2, Table 2), which matches the recent distribution of this most common tick species in most parts of Continental and Alpine biogeographic regions in Croatia [20,53], and also partly throughout Europe [57,58,59]. For instance, similar results were recorded in the area of southern England, where *I. ricinus* was represented by 96% [60] or in the Belgrade region, where it was represented with 97.4% in the collected sample [61], while in samples from 42 municipalities in Bosnia and Herzegovina *I. ricinus* was represented by 63.8% [62]. Furthermore, in Eastern Croatia *I. ricinus* was present in 72.8% [53], in green areas in the city of Osijek in 74.2%, while in the vicinity of the city of Beli Manastir, it was present in 65.9% of the collected sample [54,63]. Then, in the City of Zagreb, the only species of hard ticks, detected from 2016 to 2021 at three recreational sites was *I. ricinus*, while in three inland Croatian mountain regions—Gorski Kotar, Medvednica and Papuk—*I. ricinus* accounted for almost 99% of the tick catch between 2019 and 2021 [20,64]. The high prevalence of *I. ricinus* (99.8%) within tick population on MM indicates that existing environmental conditions support dense, but not so diverse hard tick fauna in the deciduous oak and beech forests or mixed beech–fir forests through the wide range of altitudes (200–1000 m a.s.l.) [65]. The highest overall number of hard ticks (*N* = 553) sampled at 800 m a.s.l., at MM (Table 2), indicate that microclimatic conditions of high relative humidity within layers of permanent leaf litter are particularly favorable for *I. ricinus* within deciduous beech forest, which is in line with Kahl and Gray’s [65] recent review on the ecology of this tick species. Additionally, the second altitude with the most abundant (*N* = 380) tick population on MM, was at 1000 m a.s.l., where mixed beech–fir forest maintains a suitable humid microhabitat (Table 2). This result can be explained by long-lasting saturation deficit (i.e., moisture stress) that is measured at the lower elevations, leading to higher questing tick density at higher altitudes [66,67].

Even though it was not assessed within this study, host availability at the MM study site is another important element that makes these forest communities suitable for *I. ricinus* due to the abundance and diversity of small and large wild vertebrate (Amphibia: 10; Reptilia: 11; Mammalia: 34) and bird (Aves: 96) species [68,69,70]. These hosts suit *I. ricinus*, which shows a low degree of host specificity [59,71], despite spending a significant portion of its lifespan off the host in the environment, either unfed, engorged, in developmental diapause or developing to the next stage [72].

It is known that the activity of this hard tick is regulated by photoperiod and limited to a temperature range between 5 and 30 °C, as well as favorable environments in the vegetation and leaf litter of deciduous or mixed forests with at least 80% relative humidity [71,73]. The fact that Baranja is one of the driest and warmest regions in Croatia, with the lowest amount of precipitation [24], presumably contributes to a lower abundance of the *I. ricinus* on BH compared to other areas of Continental Croatia. In addition to the influence of climate on the number of collected host-seeking specimens of *I. ricinus*, the vegetation cover also has an influence. The northeastern side of BH is covered with communities of secondary deciduous forests, and the southwestern side is covered with various agrobiocenoses, semi-natural grasslands and small remnants of forests. Despite this, an almost equal number of specimens of *I. ricinus* were collected from both sides of the BH (NE had 639 specimens; SW had 665 specimens). These results from BH are also in line with data recorded in some European urban green habitats showing that *I. ricinus* can occur in quite high numbers in gardens, parks, cemeteries and other suburban locations (especially when close to a forest) [74,75]. In summary, hard tick monitoring conducted at both of our study sites confirms the importance of *I. ricinus* as the most common, widespread and abundant tick species in the local tick fauna, which has been studied worldwide for almost a century now [76,77,78].

Comparison of hard tick faunas of four vegetation types in Bansko Hill showed small differences between them, and a similar pattern was observed on Medvednica Mountain (Table 5 and Table 6). Also, a similar pattern was observed in the surroundings of the city Beli Manastir in the Croatian part of Baranja [54].

Seasonal activities of different hard tick species collected at AHs and NHs within our research, show certain differences. On BH, bimodal seasonal activity was recorded for *I. ricinus* (Figure 3 and Figure 4). Since the temperature percentile ranks were extremely warm at BH during summer 2024 [44], recorded bimodal seasonality is in line with previous findings of Randolph et al. (2002) [79], concluding that high summer temperatures may contribute to fast development of spring-fed hard ticks, i.e., to autumnal peaks. Bimodal activity pattern was earlier described for *I. ricinus* in Croatia and Central Europe [80,81], while on MM, as well as in southern Germany, a unimodal activity pattern occurred more often [82] (Figure 5). Although increasing temperatures have been observed to contribute to the loss of seasonality, they also allow for the expansion of their altitudinal and geographic range, extending the hard-tick questing season into winter and improving the winter survival of hosts, especially deer [71,83]. In general, a unimodal activity pattern mainly occurs at northern latitudes and a bimodal one in areas with warmer and longer summers [84]. In a recent study on BH, the first peak in abundance was recorded in April and the second one in October (Figure 3 and Figure 4). In April last year, the first peak in abundance was recorded on MM, while a very light increase in number of collected hard ticks was noted during September and November 2024 at altitudes between 400 and 800 m a.s.l., but not at 200 m a.s.l., and 1000 m a.s.l., (Figure 5), which corresponds to Jouda et al. (2004) [85] reporting that at the highest altitudes no autumn peaks are usually observed when compared to lower altitudes. In previous studies in seven counties in Croatia, the first peak in abundance for *I. ricinus* was recorded a month or two later (in May and June), while the second one was recorded at the same time as in this study [81]. In Vojvodina., the northern province of the Republic of Serbia, *I. ricinus* was most abundant in May [9], similar to the data of Vilibić-Čavlek et al. (2024) [81]. In the temperate zone nymphs and adults of *I. ricinus* can be present on vegetation at all times of the year [86]. During the summer months, adults and nymphs of *I. ricinus* at BH were recorded in very small numbers and accounted for 0.61% of the collected sample of this species. These data correspond to those obtained in southern Italy, where a decline of the number of nymphs and adult *I. ricinus* ticks was also observed during the summer [87]. They were absent from BH only in August. Nymphs in the collected sample of *I. ricinus* on BH prevail at 69.09% with a peak in abundance in April, while on MM, they amount to 62.35% of collected hard ticks (Table 4). This is consistent with data obtained on the seasonal dynamics of *I. ricinus* nymphs in Ireland [88]. The large number of nymphs in the sample collected on BH and MM was probably influenced by the choice of the flag-dragging sampling method. This is a method suitable for collecting questing *I. ricinus*, especially nymphs, which are more uniformly distributed than larvae or adults [71].

The second most abundant hard tick species, collected in this study in all 12 localities at BH, was *H. inermis* (Table 2). The high proportion of *H. inermis* (42.07%) in the collected sample is most likely a consequence of optimal climatic conditions, especially the amount of annual precipitation, which in Baranja ranges between 600 and 700 mm. The optimal annual rainfall for this hard tick species is between 600 and 833 mm [89]. The highest activity peak for *H. inermis* was recorded in November (Figure 3), which is consistent with data from Central Europe when a peak of activity was also recorded in the autumn months [90]. Adults predominate in the collected sample with 99.65%, while nymphs account for 0.35%. Larvae or nymphs of *H. inermis* are rarely found in nature (on vegetation) because they feed only for a very short time [86]. These facts may explain the high proportion of adults in the sample collected on BH.

In this study, *H. concinna* was the third most abundant species after *I. ricinus* and *H. inermis* (Table 2). Also, along the Danube and Morava rivers in the Czech Republic, Slovakia, Austria and Hungary, *H. concinna* was the third most abundant species [91]. Typical habitats for *H. concinna* in Central Europe are mainly found in humid landscapes, and it has been found in forest steppe and humid steppe habitats, mainly in Asia [91]. On BH, 32.61% of *H. concinna* ticks were collected at the Branjina locality which is covered with grassland vegetation surrounded by forests similar to forest–steppe habitats. In Hungary records of *H. concinna* are more easily associated with wet habitats or forest edges [86], unlike the finds of *H. concinna* on BH. In the collected sample of *H. concinna*, nymphs predominate with 71.24%. The highest number of nymphs as well as adults was recorded in May. Maximum activity for adults in Central Europe was recorded in June, while for nymphs, it was from mid-April to mid-October [91]. Our data on seasonal activity partially overlaps with data from the literature, especially for the nymph stage. On BH, *H. concinna* was active in the period from March to July, while in Hungary in a much shorter period of time, from May to July [92].

The next two most abundant tick species are *D. marginatus* and *D. reticulatus* (Table 2). *Dermacentor marginatus* prefers areas with a warmer and drier climate, while *D. reticulatus* prefers areas with a moderately moist climate [93]. These facts are most likely the reason for the greater abundance of *D. marginatus* compared to *D. reticulatus* on BH. *Dermacentor marginatus* was often restricted to southeastern areas in some countries, mainly under hot and dry climate conditions similar to Mediterranean [94]. Similar climatic characteristics prevail on BH, giving this area sub-mediterranean climatic characteristics [25]. An equal number of *D. marginatus* specimens were collected on both sides of BH northeast and southwest. In April and May 80% of *D. marginatus* ticks were collected. These records are in accordance with the observed peak of activity for this species in Central Serbia [57], in contrast to Hungary, where the abundance of *D. marginatus* was highest in February and March [86,95]. In many parts of Europe, *D. reticulatus* is the second most abundant hard tick species after *I. ricinus*, which seem to receive growing public interest because of its potential increasing epidemiological importance [96,97,98]. According to data from previous studies in eastern Croatia, *D. reticulatus* was recorded as the third most abundant species [53] while it was recorded as first in floodplain forests in Central Posavina region in Continental Croatia [99]. Within this research at BH, it was recorded as the fifth most abundant species, and second most abundant at MM. Only 10 specimens of *D. reticulatus* ticks were collected on BH, from which 60% of specimens were collected on the Beli Manastir locality on the edge of the black locust forest (Table 1 and Table 2). This is likely due to the fact that *D. reticulatus* is not a forest inhabitant in Central Europe, but prefers more open terrains where the soil surface is more exposed to the sun in the summer than the forest floor [100]. Previously known to occur in the western Palaearctic regions with generally mild climates, this highly adaptable species recently expanded its distribution to areas of northwestern and Central Europe (Germany, Poland, Hungary, Slovakia, Netherlands, Belgium), earlier known as unsuitable (i.e., too cold) for it to survive and complete its life-cycle [93]. The data on horizontal distribution of *D. reticulatus* in Croatia so far included different AHs and NHs within the Continental biogeographic region [99,101,102], while the highest altitude till now, on which this species was sampled (around 800 m a.s.l.) included alpine bioclimatic region; Udbina (Lika-Senj County, Croatia). Within this research, two specimens of *D. reticulatus* were collected within mixed beech–fir forest at 1000 m a.s.l., which represents the first record for MM, and also for Croatia, at this altitude (Table 1 and Table 2). Our results are in line with the recent data about the range expansion of this species to higher altitudes throughout Europe, highlighting the ability of this species to tolerate notable temperature and humidity variations and inhabiting higher mountain regions, but within favorable climatic conditions [98,103,104].

In this study, *I. frontalis*, *I. hexagonus* and *I. kaiseri* tick species are each represented by only one specimen. From a medical and veterinary point of view, *I. hexagonus* is important since tick-borne encephalitis virus (TBEV) was recently isolated from this hard tick species in Croatia [105], indicating a high vector potential. The finding of *I. frontalis* adds up to previous data on its longitudinal range in Croatia, including parts of all three Croatian biogeographic regions: Continental (Zagreb), Alpine (Velebit) and Mediterranean (Istra, Dubrovnik) [102,106,107]. This is also a confirmation of recent findings where *I. frontalis* was recorded in the Kunjevci Forest Park, south of Vinkovci, which was one of the first records for this species in Eastern Croatia [108]. In Europe, this ornitophilic tick can be found in Southern, Central and Eastern part of the continent [109,110,111], where it is often collected from bird nests, directly from resident or migrating birds [112,113]. In addition to the above, our results correspond to those recorded in several regions in Germany, where *I. frontalis* was collected by the flagging method within the urban areas and gardens, indicating that this is a common tick species within AHs [114]. The latter is also partly in contrast to our results, since we had only one catch of *I. frontalis* at AHs and one at NHs (Table 2). The same author points out that the Eurasian blackbird (*Turdus merula*) represents one of the most common bird hosts for *I. frontalis*, while previous reports from our country mention bohemian waxwing (*Bombycilla garrulus* L.) and black redstart (*Phoenicurus ochruros*) as hosts [107,115]. Further on, the fact that we collected *I. frontalis*—known as an ubiquitous bird parasite [110]—at the MM site as well as at BH, is in line with the richness of ornitofauna (cca 96 bird species) found at Nature Park Medvednica [22]. These updated information on the range of *I. frontalis* in Croatia can contribute to improving public health protection, since this tick species is known as a potent vector of *Borrelia turdi* [110] and is also suspected to cause the avian-related syndrome in its hosts [116].

Having in mind that hard-tick questing activity can considerably vary throughout the species distribution and also that is it depends of numerous factors (e.g., air temperature, humidity, light, host species, abundance, behaviors, vegetation, etc.), we evaluated the questing hard tick density (i.e., tick phenology) dependent on the weather conditions [117,118]. The average annual air temperatures in Croatia—for years 2019, 2020, 2021, 2023 and 2024—were above the multi-annual average and the precipitation varied (from dry to wet at MM, and from wet to normal at BH) when compared to the 30-year reference climate period (1981–2010: before 2023, 1991–2020: after 2023) [44,45].

At anthropogenic habitats at BH, a significant (*p* < 0.05) negative correlation was determined between the abundance (*N*) and the mean monthly air temperatures (°C) for *H. inermis* (*r* = −0.5931; *p* = 0.0421) and *D. reticulatus* (*r* = −0.6289; *p* = 0.0285), while their numbers positively correlated (*r* = 0.5551; *p* = −0.2667; *r* = 0.4430; *p* = 0.1492) with the air humidity (%) (Table 7). These results correspond to a previously known activity pattern, seen for *D. reticulatus*, that is more strongly determined by temperature than humidity, and to a specific seasonal dynamics and the highest activity peaks for those two species, seen during autumn months [90].

Over a three-year period, the highest overall number of hard ticks at NH on MM was recorded in 2020 (Figure 5), when the thermal conditions were very warm, but the precipitation was normal compared to multi-annual average, confirming that overall rainfall (thus humidity) is an important (crucial) factor in tick survival, i.e., high densities [118].

The number of sampled host-seeking *I. ricinus* ticks (♀, ♂, nymph, larva) at natural forest habitats on MM was positively associated with air temperature (°C) and negatively with air humidity at elevations from 200 to 1000 m a.s.l. (*r* = −0.7684; *p* = 0.0259; at 200 m a.s.l.).

In anthropogenic habitats at BH, which have an open, heterogeneous tree canopy and higher sun exposure, the increased activity and abundance of host-seeking ticks (∑ Ixodidae) are negatively correlated with air temperature and positively correlated with air humidity (Table 7). This suggests that ticks in these open environments are more active during cooler, more humid periods to mitigate the risk of desiccation [119]. Conversely, in homogenous mountainous forests on MM, the higher activity of *I. ricinus* ticks is positively correlated with higher air temperatures and negatively correlated with air humidity (Table 8). The stable, humid microclimate provided by the dense canopy likely allows these ticks to remain active even when the external air temperature rises and humidity drops.

These results are consistent with the biological traits of hard ticks, particularly *I. ricinus* and its sensitivity to humidity [119]. While ticks need to maintain water balance to prevent desiccation [120,121,122,123], the richness and the structure of hard tick fauna, and also their activity periods, are influenced by (micro)climatic conditions, associated with the habitat type (Figure 2), especially the canopy cover.

Numerous hard tick species are vectors of many pathogens that threaten human and animal health (e.g., tick-borne encephalitis virus, *Borrelia burgdorferi* s.l., *B. miyamotoi*, *Rickettsia* spp., *Anaplasma phagocytophilum*, *A. marginale*, *Babesia divergens*, *B. microti*, *B. canis*, *B. caballi*, *Francisella tularensis*, *Coxiella burnetii*, *Theileria equi*) [124,125,126,127].

Taking into consideration that medically and economically relevant hard tick species, like the ones recorded within this study (*I. ricinus*, *D. reticulatus*, *D. marginatus,* etc.), will possibly benefit from changes in human activities, agricultural use, animal trade and traveling, reforestation, wildlife population growth, climate changes, etc., [128,129,130], and expand their range and abundance increasing the risk of tick-borne diseases at the same time [103], data on the distribution, abundance and the seasonality of tick species will, beyond doubt, remain highly important [98,131].

Our study offered data on seasonal and monthly dynamics of eight different hard tick species and their developmental stages at two study sites in relation to weather anomalies and habitat types, emphasizing the importance of local conditions to hard tick density and abundance. It also offers new data regarding longitudinal range of *I. frontalis* and altitudinal range of *D. reticulatus*, both medically and economically relevant tick species. Such studies are important in identifying high-risk periods and risk magnitudes and are also useful for the occasional or professional land users in terms of preventing hard tick bites, i.e., tick-borne diseases. Systematic, interdisciplinary and empirical local studies are needed for better understanding the complex interactions between numerous ecological conditions and human exposure to tick-borne pathogens.

## 5. Conclusions

This is the first report on the comparison of hard tick (Ixodidae) fauna between natural habitats (NHs) (i.e., forest communities) and anthropogenic habitats (AHs) (i.e., orchards, grasslands, degraded forests) in Croatia.

Eight tick species (*D. marginatus*, *D. reticulatus*, *H. concinna*, *H. inermis*, *I. frontalis*, *I. hexagonus*, *I. kaiseri* and *I. ricinus*) were recorded in AHs on Bansko Hill (BH), Eastern Croatia, while only three species (*I. ricinus*, *I. frontalis*, *D. reticulatus*) were identified in NHs on Medvednica Mountain (MM), Central Croatia.

In both studied areas *I. ricinus* was the most abundant species. Nymphs in the collected *I. ricinus* sample prevailed in both studied areas. Most hard ticks were collected in spring in both study areas. On BH a bimodal activity pattern was recorded for *I. ricinus* and *H. inermis*, while a unimodal activity pattern was recorded for *I. ricinus* on MM. The comparison of hard tick fauna in different habitats using the Sørenson index on BH and MM showed a high percentage of similarity (50.0–88.8).

A correlation analysis showed that in more exposed, heterogeneous anthropogenic habitats, the increased activity of sampled host-seeking hard tick species (∑ Ixodidae) is linked to lower air temperatures and higher humidity, while in dense, homogeneous mountainous forests, the higher activity of *I. ricinus* ticks is associated with higher air temperatures and lower humidity. Both of these results are consistent with the biological characteristics of ticks, since they need to maintain water balance in a drier environment and avoid desiccation.

The finding of *I. frontalis* represents the first record of this tick species for BH and Osijek–Baranja County, while the specimens of *D. reticulatus* collected on Medvednica Mountain, represent the first catch at 1000 m a.s.l. in Croatia.

Overall, our findings provide new data on the seasonality, horizontal and vertical distribution of medically and economically important tick species in Continental Croatia. This information, analyzed in relation to weather anomalies and habitat types, contributes to identifying tick-risk foci and high-risk periods.

## Figures and Tables

**Table 1:** List of tick sampling sites with coordinates and altitudes.

Location	Tick Sampling Site	Altitude, Latitude (Degree/min/s)	Altitude (m a.s.l.)	Habitat Type
Bansko Hill	01 Batina	N 45.854651 E 18.849288	128	Untended orchards
	02 Beli Manastir	N 45.767953 E 18.625718	129	Black locust degraded forest
	03 Branjina	N 45.820343 E 18.699563	86	Grassland with black locust and walnut on the edges
	04 Draž (Vidikovac Trojnaš)	N 45.826566 E 18.805027	194	Untended orchards
	05 Kamenac (Odašiljač Belje)	N 45.796069 E 18.691250	239	Black locust degraded forest
	06 Karanac (Vidikovac Belje)	N 45.775711 E 18.669302	199	Black locust degraded forest
	07 Kotlina	N 45.788050 E 18.737783	115	Mixed degraded forest
	08 Kneževi Vinogradi	N 45.762711 E 18.716909	105	Untended orchards
	09 Podolje	N 45.816064 E 18.728725	97	Untended orchards
	10 Popovac (Rudnik)	N 45.784287 E 18.662404	231	Black locust degraded forest
	11 Suza	N 45.794460 E 18.741029	138	Mixed degraded forest
	12 Zmajevac	N 45.800027 E 18.798309	122	Mixed degraded forest
Medvednica mountain	13 Medvednica 200 m a.s.l.	N 45 51.174 E 16 01.172	229	Pedunculate oak forest with European hornbeam ^1^
	14 Medvednica 400 m a.s.l.	N 45 52.748 E 15 58.568	422	European beech forest ^2^
	15 Medvednica 600 m a.s.l.	N 45 53.201 E 15 58.330	611	European beech forest ^2^
	16 Medvednica 800 m a.s.l.	N 45 53.394 E 15 57.634	810	European beech forest ^2^
	17 Medvednica 1000 m a.s.l.	N 45 53.916 E 15 56.753	1003	Pannonian beech–fir forest ^3^

^1^ Pedunculate oak forest with European hornbeam (*Carpino betuli—Quercetum roboris typicum* Rauš 1969), ^2^ European beech forest with Woodruff (*Galio odorati-Fagetum* Sougnez et Thill 1959), ^3^ Pannonian beech–fir forest (*Festuco drymeiae—Abietetum* Vukelić et Baričević 2007).

**Table 2:** Number (*N* %) of collected ticks, depending on the species and sampling locality.

Species/ Locality	*Ixodes* *ricinus* *N*; %	*Haemaphysalis inermis* *N*; %	*Haemaphysalis concinna* *N*; %	*Dermacentor* *marginatus* *N*; %	*Dermacentor* *reticulatus* *N*; %	*Ixodes* *frontalis* *N*; %	*Ixodes* *hexagonus* *N*; %	*Ixodes* *kaiseri* *N*; %	Σ Σ *N*; %
Popovac	232; 41.7	287; 51.6	22; 4.0	14; 2.5	1; 0.2	0; 0.0	0; 0.0	0; 0.0	556; 13.0
Zmajevac	234; 64.3	97; 26.6	29; 8.0	0; 0.0	2; 0.5	1; 0.3	1; 0.3	0; 0.0	364; 8.5
Branjina	207; 57.3	77; 21.3	76; 21.1	1; 0.3	0; 0.0	0; 0.0	0; 0.0	0; 0.0	361; 8.5
Karanac	91; 25.3	222; 61.8	43; 12.0	3; 0.8	0; 0.0	0; 0.0	0; 0.0	0; 0.0	359: 8.4
Beli Manastir	62; 19.6	227; 71.8	11; 3.5	10; 3.2	6; 1.9	0; 0.0	0; 0.0	0; 0.0	316; 7.4
Suza	152; 53.1	95; 33.2	38; 13.3	0; 0.0	0; 0.0	0; 0.0	0; 0.0	1; 0.3	286; 6.7
Draž	170; 92.9	13; 7.1	0; 0.0	0; 0.0	0; 0.0	0; 0.0	0; 0.0	0; 0.0	183; 4.3
Podolje	30; 29.1	65; 63.1	7; 6.8	0; 0.0	1; 1.0	0; 0.0	0; 0.0	0; 0.0	103; 2.4
Kotlina	65; 69.9	27; 29.0	0; 0.0	1; 1.1	0; 0.0	0; 0.0	0; 0.0	0; 0.0	93; 2.2
Kamenac	14; 36.8	20; 52.6	3; 7.9	1; 2.6	0; 0.0	0; 0.0	0; 0.0	0; 0.0	38; 0.9
Kneževi Vinogradi	21; 61.8	9; 26.5	4; 11.8	0; 0.0	0; 0.0	0; 0.0	0; 0.0	0; 0.0	34; 0.8
Batina	26; 78.8	7; 21.2	0; 0.0	0; 0.0	0; 0.0	0; 0.0	0; 0.0	0; 0.0	33; 0.8
**Σ = 12**	1304; 47.8	1146; 42.0	233; 8.5	30; 1.1	10; 0.4	1; 0.04	1; 0.04	1; 0.04	2726; 100.0
Medvednica (200 m a.s.l.)	85; 98.8	0; 0.0	0; 0.0	0; 0.0	0; 0.0	1; 1.2	0; 0.0	0; 0.0	86; 2.0
Medvednica (400 m a.s.l.)	227; 100.0	0; 0.0	0; 0.0	0; 0.0	0; 0.0	0; 0.0	0; 0.0	0; 0.0	227; 5.3
Medvednica (600 m a.s.l.)	297; 100.0	0; 0.0	0; 0.0	0; 0.0	0; 0.0	0; 0.0	0; 0.0	0; 0.0	297; 7.0
Medvednica (800 m a.s.l.)	553; 100.0	0; 0.0	0; 0.0	0; 0.0	0; 0.0	0; 0.0	0; 0.0	0; 0.0	553; 13.0
Medvednica (1000 m a.s.l.)	378; 99.5	0; 0.0	0; 0.0	0; 0.0	2; 0.5	0; 0.0	0; 0.0	0; 0.0	380; 8.9
**Σ = 5**	1540; 99.8	0; 0.00	0; 0.00	0; 0.00	2; 0.01	1; 0.01	0; 0.00	0; 0.00	1543; 100.00
**Σ Σ = 17**	2844; 66.62	1146; 26.84	233; 5.46	30; 0.70	12; 0.28	2; 0.05	1; 0.02	1; 0.02	4269; 100.00

**Table 3:** Species list, sex ratio and developmental stages of hard ticks collected on Bansko Hill (2023–2024).

Species/Developmental Stage	Females *N*; %	Males *N*; %	Nymphs *N*; %	Larvae *N*; %	Total *N*; %
*Ixodes ricinus*	172; 13.2	103; 7.9	901; 69.1	128; 9.8	1304; 47.8
*Haemaphysalis inermis*	828; 72.3	314; 27.4	4; 0.3	0; 0.0	1146; 42.0
*Haemaphysalis concinna*	10; 4.3	18; 7.7	166; 71.2	39; 16.7	233; 8.5
*Dermacentor marginatus*	21; 70.0	9; 30.0	0; 0.0	0; 0.0	30; 1.1
*Dermacentor reticulatus*	8; 80.0	2; 20.0	0; 0.0	0; 0.0	10; 0.04
*Ixodes frontalis*	1; 100.0	0; 0.0	0; 0.0	0; 0.0	1; 0.04
*Ixodes hexagonus*	1; 100.0	0; 0.0	0; 0.0	0; 0.0	1; 0.04
*Ixodes kaiseri*	1; 100.0	0; 0.0	0; 0.0	0; 0.0	1; 0.04
**∑ = 8**	1042; 38.22	446; 16.36	1071; 39.29	167; 6.13	2726; 100.00

**Table 4:** Species list, sex ratio and developmental stages of hard ticks collected on Medvednica Mountain (2019–2024).

Species/Developmental Stage	Females *N*; %	Males *N*; %	Nymphs *N*; %	Larvae *N*; %	Total *N*; %
*Ixodes ricinus*	107; 6.9	148; 9.6	961; 62.4	324; 21.0	1540; 99.8
*Dermacentor reticulatus*	1; 50.0	1; 50.0	0; 0.0	0; 0.0	2; 0.1
*Ixodes frontalis*	0; 0.0	0; 0.0	1, 100.0	0; 0.0	1; 0.1
**∑ = 3**	108; 7.0	149; 9.7	962; 62.3	324; 21.0	1543; 100.00

**Table 5:** Sørenson index values for tick faunas in different vegetation type on Bansko Hill.

Vegetation Type	Black Locust Degraded Forests	Mixed Degraded Forests	Untended Orchards	Semi-Natural Grasslands
Black locust degraded forests	-	76.92	88.88	88.88
Mixed degraded forests	-	-	66.66	66.66
Untended orchards	-	-	-	75

**Table 6:** Sørenson index values for tick faunas in different vegetation type on Medvednica Mountain.

Vegetation Type	Pannonian Beech–Fir Forests	European Beech Forests	Pedunculate Oak Forest with European Hornbeam
Pannonian beech–fir forests	-	66.66	50.00
European beech forests	-	-	66.66

**Table 7:** Correlation (*r* = Pearson’s correlation coefficient) between monthly climate parameters (temperature and relative humidity) and hard tick species abundance (*N*) in AHs at Bansko Hill (January–December 2024).

Species	Temperature (°C)		Relative Humidity (%)	
	*r* *	*p*-Value	*r*	*p*-Value
*Ixodes ricinus*	−0.1016	0.7533	−0.2538	0.4261
*Haemaphysalis inermis*	−0.5931	0.0421	0.5551	0.0610
*Haemaphysalis concinna*	0.1910	0.5521	−0.2667	0.4020
*Dermacentor marginatus*	0.1216	0.7065	−0.3826	0.2197
*Dermacentor reticulatus*	−0.6289	0.0285	0.4430	0.1492
*Ixodes frontalis*	0.0151	0.9628	−0.3590	0.2518
*Ixodes hexagonus*	0.1594	0.6206	−0.0531	0.8697
*Ixodes kaiseri*	0.0151	0.9628	−0.3590	0.2518
∑ Ixodidae	−0.4518	0.1403	0.2143	0.5037

*r* * = Pearson’s correlation coefficient.

**Table 8:** Correlation (*r* = Pearson’s correlation coefficient) between the weather conditions (air temperature (°C), relative air humidity (%)) and the *I. ricinus* abundance (*N*) for different development stages (♀, ♂, *N* = nymph, L = larva) and altitudes (200–1000 m a.s.l.) in NHs on Medvednica Mountain (April–December 2024).

*I. ricinus* Stages	Temperature (°C)		Relative Humidity (%)		*I. ricinus* m a.s.l	Temperature (°C)		Relative Humidity (%)	
	*r* *	*p*-Value	*r*	*p*-Value		*r* *	*p*-Value	*r*	*p*-Value
♂	0.1646	0.6968	−0.5137	0.1928	200	0.2028	0.6301	**−0.7684**	**0.0259**
♀	0.1692	0.6887	−0.5439	0.1635	400	0.0776	0.8552	−0.6473	0.0827
N	0.1887	0.6544	−0.4731	0.2364	600	0.2156	0.6081	−0.3921	0.3366
L	0.4780	0.2308	−0.3515	0.3931	800	0.0255	0.9523	−0.4344	0.2822
Ʃ IR	0.2099	0.6178	−0.5149	0.1916	1000	0.3793	0.3541	−0.5614	0.1476

*r* * = Pearson’s correlation coefficient.

## Data Availability

The data presented in this study are available on request from the corresponding author.
